# Optimization of an ecological integrity monitoring program for protected areas: Case study for a network of national parks

**DOI:** 10.1371/journal.pone.0202902

**Published:** 2018-09-19

**Authors:** Jérôme Théau, Simon Trottier, Patrick Graillon

**Affiliations:** 1 Department of Applied Geomatics, Université de Sherbrooke, Sherbrooke, Québec, Canada; 2 Société des établissements de plein air du Québec (Sépaq), Québec city, Québec, Canada; Swedish University of Agricultural Sciences and Swedish Institute for the Marine Environment, University of Gothenburg, SWEDEN

## Abstract

Over the last few years, several of the world’s national park networks have implemented ecological integrity monitoring programs. These programs are based on a series of indicators to detect changes in ecosystem integrity. There are many scientific and logistical challenges in developing these programs due to limits in both our knowledge of ecosystems functioning and the resources for implementing such programs. Thus, the relatively quick and simple implementation of many monitoring programs has been to the detriment of their scientific validity. Few studies have focused on evaluating an entire monitoring program. This project presents an approach to evaluate the ecological and statistical relevance of ecosystem integrity indicators measured within a program with the goal of iterative optimization. The approach is based on three complementary elements: (1) spatial characterization of park ecosystems based on the classification of satellite imagery, (2) ecological validation of indicators based on ecosystem conceptual models and (3) statistical validation of indicators based on analyses of statistical power. This innovative approach allows a systematic, quantified, reproducible and generalizable review of the indicators of an ecological integrity monitoring program. It provides managers with an overview of the spatial representativeness of indicators, their ecological and statistical relevance according to different parameters such as the period monitored, the amount of change to be detected, and the degree of significance. Thus, the approach identifies monitoring gaps and offers various alternatives for improving sampling. The approach was developed and tested in the network of Quebec national parks, more specifically in the Frontenac, Jacques-Cartier and Bic national parks. The results clearly identify the strengths and weaknesses of the current program in place and possible improvements are proposed for these parks. This approach is a relevant tool for park networks, particularly for those that have limited resources for monitoring ecological integrity.

## 1. Introduction

Worldwide, national parks are part of the solution for biodiversity loss [1]. However, with the rise in popularity of parks over the last few decades, managers are wondering about the possible effects of increasing anthropogenic pressures on ecosystems. In some cases, managers are also facing pressure from the scientific community and the public to show that they can properly manage national parks [2],[3]. Establishing an ecological monitoring program is an option chosen by many networks to monitor the state of parks. These programs are characterized by the use of indicators that follow changes in ecological integrity. Ideally, it must (1) be ecologically relevant, (2) allow the detection of anthropogenic changes and their intensity, (3) be based on sound statistics, (4) have a favourable cost-benefit ratio and (5) be understandable to managers, scientists, and the population [4]-[6]. Many ecological monitoring programs are in place or in development in national parks around the world [6]. However, these programs face many conceptual and methodological challenges.

There is a lack of consensus in the scientific community regarding the measurement of ecological integrity. One of the basic problems is identifying the baseline conditions, which can be absolute or relative. An absolute baseline refers to ecosystems that are not affected by any significant anthropogenic pressures. For North America, this generally corresponds to the conditions before massive European colonization [4]. However, determining these baseline conditions remains difficult; the ecosystems need to have been subject to sufficient scientific studies to document their initial state and evolution since that time [6]. A relative baseline is easier to apply and involves considering ecological integrity as a gradient of human influence on natural environments. The less anthropogenic pressure on an ecosystem, the higher it is on the gradient. Thus, an ecological monitoring program uses as a baseline the ecosystem state at time *t* and measures the direction and magnitude of changes in this state over time. Determining whether there has been an improvement or degradation of the level of ecological integrity involves using fixed change thresholds. But, determining these thresholds based on accurate scientific knowledge is another challenge in ecological monitoring [7],[8].

The selection of ecological integrity indicators is often based on the use of ecosystem conceptual models [2],[8]-[10]. These models identify and describe anthropogenic stress, natural disturbances and key ecological components as well as interactions between these elements for a given ecosystem [2],[8]. Thus, this approach prioritizes the ecological aspect regardless of the economic and practical aspects, which are too often prioritized when choosing indicators [11], but it remains relatively subjective and dependent on the developer’s point of view [12].

Efficiently measuring an ecosystem’s ecological integrity requires that the selected indicators represent all attributes and scales of this ecosystem [4],[13]. Thus, the difficulty is to choose a set of indicators that correctly represents ecological integrity and the complexity of a national park’s ecosystems while being consistent with the monitoring program’s objectives and the resources available to managers [4],[13].

The possibility of drawing real and scientifically defensible conclusions from the measurement of indicators depends on the quality of the sampling protocols [10],[14]. However, the rigorous application of sampling methods is often associated with relatively high costs, which causes some managers to neglect this aspect [5],[15]. Three elements usually characterize a sampling design: sampling location, number of samples and sampling frequency [10]. Recent statistical studies present increasingly sophisticated sampling methods that adapt to the constraints and various situations faced by managers of ecological monitoring programs [16].

In most cases, managers will use power analysis to address the concept of the number of samples [5],[8],[9]. This statistical measure helps to determine the number of samples required to detect a change in an indicator’s measurements [5].

Due to the many conceptual and methodological challenges, monitoring in protected area networks has often relied on a relatively quick and simple implementation, mainly related to the available financial and human resources [17]. These choices have allowed a relatively fast deployment of monitoring but include deficiencies that could limit their scientific validity.

**Fig 1:**
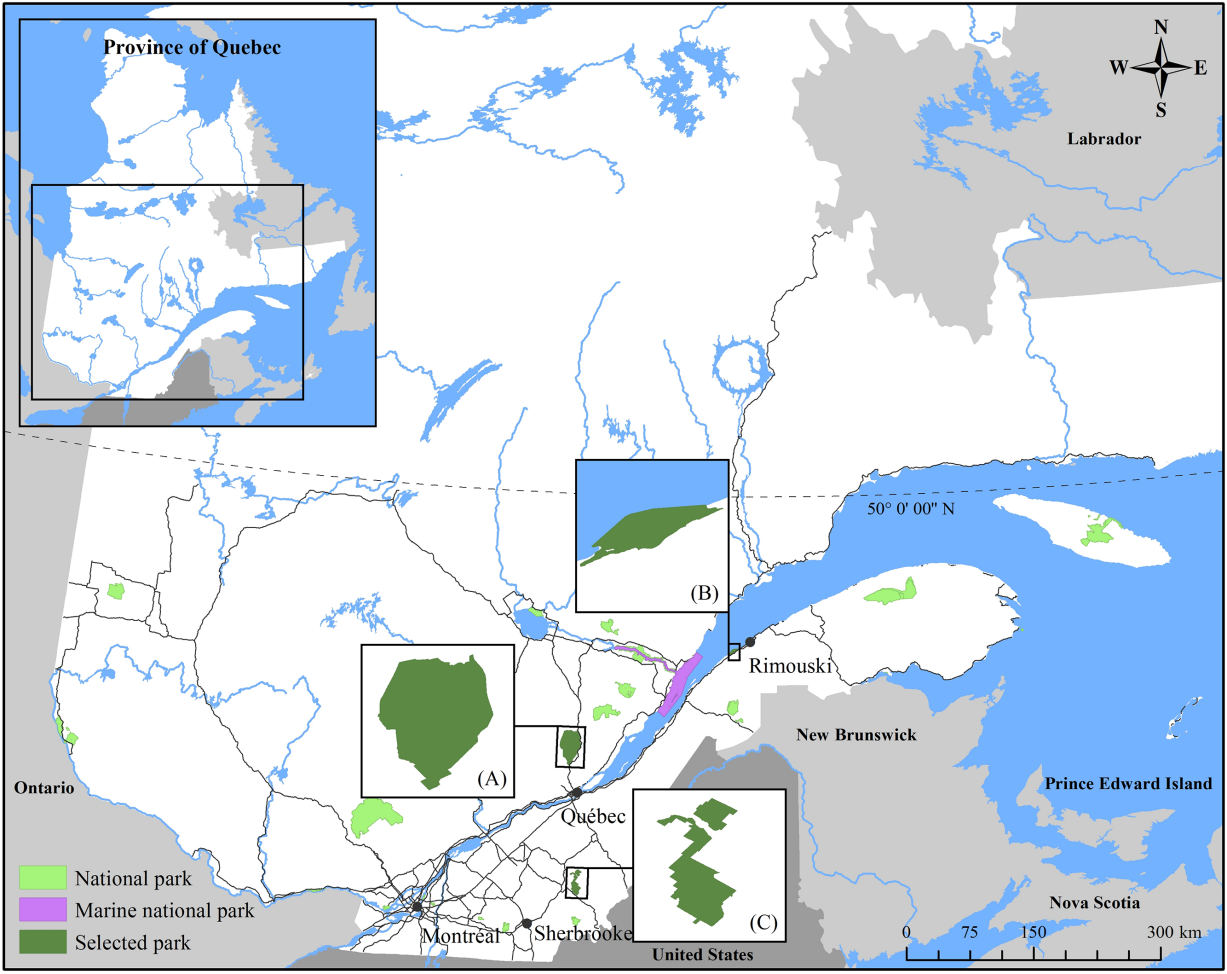
Network of the Quebec national parks included in the ecological monitoring program and parks selected for this study: (A) Jacques-Cartier, (B) Bic, and (C) Frontenac.

This has occurred in the network of national parks in Quebec, Canada (Fig 1), which participates in an ecological monitoring program since 2004: The Ecological Integrity Monitoring Program (*Programme de Suivi de l’Intégrité Ecologique*, [PSIE–in French]). This network of national parks falls under the Québec government’s jurisdiction and is managed by a government organization called the *Société des établissements de plein air du Québec* (Sépaq).

The PSIE uses a set of about 20 network indicators, as well as others that are more adapted to local ecosystem conditions for each of the 22 monitored parks [18]. The network indicators use a common proven methodology for all the monitored parks. They identify the more global conservation issues, and allow the officials of the various monitored parks to collaborate and compare results. The local indicators primarily target conservation issues specific to parks and are usually developed by park officials. Indicators are grouped into two components (ecosystem and human) and five parameters (air quality, water quality, status of biocenosis, landscape spatial organization, and infrastructure quality). The PSIE measures relative changes in ecological integrity compared to 2004, the reference year.

When the PSIE was implemented, Sépaq managers prioritized the selection of simple indicators that suited their ressources and scientific capacities. In so doing, some classic steps in setting up this kind of monitoring program were not documented with the scientific rigour usually required for this type of program. Over the last few years, the Sépaq managers have started a process to address some of these PSIE gaps, which were caused by the implementation approach recommended at the time. Thus, Sépaq managers wish to verify the extent to which the implemented indicators are representative of the parks’ ecosystems and to validate if the sampling is sufficient. They also wish to limit the loss of existing data by improving sampling, without discarding all the inherited data.

The objective of this study was to develop and test a diagnostic approach for an existing ecological monitoring program by evaluating the ecological, spatial and statistical relevance of currently followed indicators. Based on a case study in Québec national parks, this study intends to develop a systematic approach designed to be included in an iterative optimization process and transferable to other protected area networks.

## 2. Materials and methods

### 2.1 Study area

Three national parks in the network followed by PSIE were used in this study: Bic, Frontenac and Jacques-Cartier (Fig 1). These three parks were chosen for their ecosystem diversity that provides a representative sample of the entire network [19]. Du Bic Park covers 33.2 km^2^, of which 14.4 km^2^ is a marine ecosystem. It was created in 1984 to protect a representative sample of the south coast of the St. Lawrence Estuary. The Frontenac Park covers 155 km^2^; it was created in 1987 to preserve a representative site of the natural region of the hills of the Eastern Townships, Beauce and Bellechasse. It includes some ecologically important wetlands. The Jacques-Cartier Park covers 670 km^2^, mostly forest. It was created in 1981 and preserves a representative part of the Laurentian Mountains.

### 2.2 Methodological approach

The proposed methodology is divided into three distinct axes: (1) spatial characterization of ecosystems, (2) ecological validation of indicators and (3) statistical validation of indicators. Together, these three axes provide the basis of the indicator assessment for the optimization of the Ecological Integrity Monitoring Program (*Bilan des Indicateurs pour l’Optimisation du Programme de Suivi de l’Intégrité Ecologique* [BIOPSIE—in French]). BIOPSIE is a key step in the iterative optimization process for ecological integrity monitoring because it leads to the development of a diagnostic for the measured indicators and allows making recommendations to modify these indicators. This article is focused particularly on this step of the process.

#### 2.2.1 Datasets

Vector and matrix data are used in this project. The vector data are from Sépaq and are related to park boundaries, infrastructure, the location of sampling sites and data from the Quebec topographical database (*Base de données topographiques du Québec*, [BDTQ–in French]). The matrix data are SPOT 4 (HRVIR sensor) and 5 (HRG sensor) satellite images and aerial photography (S1 Table)

#### 2.2.2 Spatial characterization of ecosystems

The spatial characterization of ecosystems consists of mapping the various park ecosystems to evaluate their sampling location and their relative importance. This step ensures the acquisition of uniform ecosystems knowledge for all the parks in the study. This characterization was carried out by an unsupervised classification of multispectral SPOT 4 (HRVIR sensor) and 5 (HRG sensor) satellite images using the K-means algorithm. The nomenclature used in the classification was inspired by that of [8],[9] and includes the following classes: (1) forest (including open forests), (2) freshwater, (3) wetland, (4) built-up and (5) bare ground, which correspond to the main ecosystems in the parks studied. For Bic Park, the coastal and marine classes were also mapped. The classification results were validated using aerial photography (Bic National Park; 24 points per class) and field data acquired in 2012 (Frontenac and Jacques-Cartier national parks; 30 points per class).

#### 2.2.3 Ecological validation of indicators

The ecological validation of indicators is based on using ecosystem conceptual models and associated analysis grids. A conceptual model was used for each of the parks’ ecosystems, corresponding to classes established during the preceding step. Since the development of an ecosystem conceptual model is complex and potentially subjective [12], existing conceptual models developed by Parks Canada have been adapted. These models, created by scientific teams at Parks Canada after thorough analyses, were considered robust and applicable to Quebec national parks.

The conceptual model used for the freshwater ecosystem is presented in S1 Fig. Four types of entities are in the conceptual models [20]:

1. Stressors: anthropogenic change agents from inside or outside the parks, which may affect natural disturbances, interaction processes, and ecological components at various spatial and temporal scales (e.g. climate changes, water pollution).
2. Natural disturbances: natural change agents that can be cyclic (e.g. hydrological events), relatively predictable (e.g. temperature and precipitation) or unpredictable (e.g. fires). They can have significant effects on ecological components and linking processes, and act at various spatial and temporal scales.
3. Ecological components: they correspond to the substrate (e.g. water), trophic level (e.g. primary consumers, decomposers), a species, a group of species or any other element of interest.
4. Linking processes: they correspond to a modification, use or any other process linking a stressor, a natural disturbance, or an ecological component (e.g. predation, herbivory).

**Fig 2:**
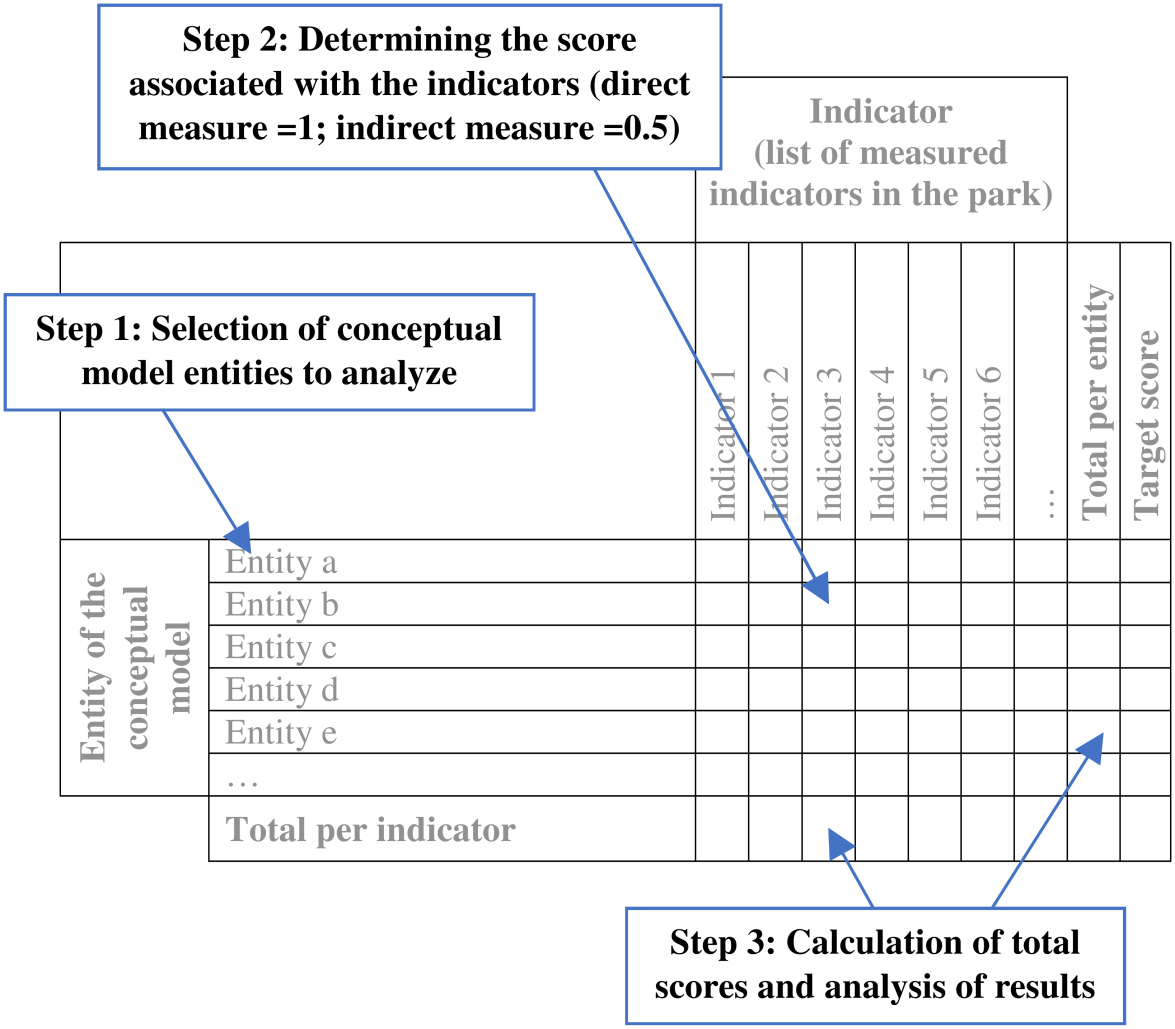
Schematization of the analysis grid for the ecological validation of indicators.

The next step is to create analysis grids to translate the information in the conceptual models to produce an ecological validation of the indicators. These grids are designed to link the information in the conceptual models with that from the indicators monitored in each park and to quantify the coverage of the various entities of the models. The design of the analysis grids was carried out in three steps (Fig 2):

1. Selection of conceptual model entities to analyze: only the stressors and ecological components were selected. Although the role of natural disturbances is essential, the PSIE indicators do not cover them as park managers cannot change their management practices in response to changes in these entities (e.g. temperature regime). As for linking processes, they are often similar to the ecological components to which they are connected (e.g. the “mortality” process vs the “secondary consumers” ecological component: monitoring an indicator linked to secondary consumers (e.g. invertebrates) allows indirect monitoring of their mortality). This selection aims to simplify the analysis grids and their use.
2. Determining the score associated with the indicators: This step is used to associate a value to the indicators for each of the selected model entities (Fig 2) by answering the following question: does indicator X allow entity Y to be measured directly or indirectly? Analyzing the strength of relationships between indicators and ecological components using weight-based approaches has been used in several studies [11],[21],[22]. As an example, [22] used a weighting process by attributing three weight levels (1; 0.67; 0.33) based on expert evaluation to quantify the strength of the relationships (high, medium and low) between ecological components and measured indicators, respectively. A similar approach based on two levels of strength was selected is this study. A value of 1 was attributed to a direct measure of the entity by the indicator and a value of 0.5 to an indirect measure. A multidisciplinary group composed of three to six experts, depending on the ecosystem, carried out this step. This simple approach was chosen because of the limited knowledge available to precisely characterize these relationships. It also allows a high repeatability of the scoring process across the park network.
3. Calculation of total scores and analysis of results: The last step is used to calculate the total score for each measured indicator and each entity of the conceptual model. Total scores are calculated for each line (i.e. each ecosystem entity) and for each column (i.e. each measured indicator) by summing numbers attributed at step 2 (Fig 2). The total score per entity corresponds to the number of measurements made for each of them. The total per indicator is used to represent and compare their comprehensiveness for measuring the various entities of the ecosystem’s conceptual model.

In this study, we set a total target score of 2 per entity. This value corresponds to the equivalent of two direct measurements of the entity by indicators. Thus, an ecosystem entity is considered well covered when a modification of its condition is reflected in more than one indicator. Even if complementarity and parsimony usually guide the selection of indicators in a monitoring program, a level of redundancy between indicators is unavoidable and even necessary. This redundancy should be limited to avoid monitoring bias and resource waste associated with an over-coverage of specific entities [23],[24]. However, redundancy can also be beneficial since the response of an ecosystem entity to a driver can vary across scales and ecosystems [24]. Moreover, indicators covering the same ecosystem entity can be sensitive to different environmental factors and should then be considered as complementary [23]. Thus, this target value allows to evaluate the level of coverage of the entities in a conservative manner while aiming at an optimization of the available resources.

It is important to mention that this target was selected in collaboration with managers of the national park network. It is closely related to their management goals and could be different and modified in another park network setting (e.g. a critical ecosystem entity could require a particular attention and a higher score target). In this case, this value should not be considered as a fixed value in the proposed approach. An evaluation of each entity relative to all the measurements and their target score was calculated. A positive evaluation indicates an entity deemed adequately measured. In other words, the indicators currently used by the PSIE allow to gather sufficient information on that entity. However, an evaluation that is too high may indicate that too much effort is invested in an entity and that these resources could be invested in another, less represented, entity. Conversely, a negative balance may indicate that an entity is not adequately considered by the indicators in place. By summing the total for each ecosystem in the park, it is also possible to analyze the relative contribution of the indicators at the scale of the park.

#### 2.2.4 Statistical validation of indicators

The statistical power calculation was applied to all the indicators for one sampling. Certain indicators, such as those measuring spatial phenomena over the entire park’s territory (e.g. fragmentation) or accurate populations for a specific site (e.g. exceptional forest ecosystems), are not suited to this type of analysis. Finally, the number of indicators for which the statistical power was calculated was 10 for Bic National Park, 11 for Frontenac and 8 for Jacques-Cartier. They are all indicators monitored by a repeated protocol. More precisely, this means that they are permanent sampling sites and are monitored at an inter- and intra-annual sampling frequency determined in advance by managers. Sampling characteristics of these indicators for Frontenac National Park are described in Table 1.

**Table 1:** Sampling characteristics of the Frontenac National Park indicators selected for statistic validation.

Parameter	Indicator	Unit	Change rate to detect	Sampling frequency (years)	Number of permanent sites	Intra-annual revisit frequency
Water quality	Benthic fauna quality	Indicator [1, 20]	0,2	3	2	1
	Lake trophic level	Visibility (m)	2%	1	8	6
	Phosphorus concentration	Mg P/L	2%	1	9	6
	Fecal coliform concentration	UCF / 100 ml	2%	1	9	6
Status of biocenosis	Non-indigenous plant propagation	Relative frequency of presence	5%	2	58	1
	Avian monitoring	Number of species per station	2%	5	50	2
	Anuran listening route	Modified Shannon-Weaver indicator	2%	1	20	3
	Ichthyological quality	Indicator [-100, 200]	1	3	4	2
	Lacustrine habitat monitoring	Number of species per station (max = 13)	0,13	3	9	3
Infrastructure quality	Hiking trail width	Width (cm)	2%	1	6	1
	Campsite quality	Indicator [0, 10]	0,1	1	50	1

The calculation of the statistical power of indicators was carried out with Monitor software [25]. This open-source software, already used by some managers of large networks such as NPS, is relatively simple to use, which is a considerable advantage for the deployment of the tool among managers who are potentially not experts in statistical analyses. The calculation of the statistical power requires the configuration of several parameters such as the number of sampled permanent sites, the parameter value at the start of monitoring, the measured variance, the inter- and intra-annual sampling frequency, the change to be detected (%) and the degree of significance (α). The power values were calculated for periods of 10 and 20 years, for detected changes of 2% and 5% and a degree of significance of 0.1 and 0.2. The inter- and intra-annual revisit values correspond to those defined in the sampling protocols for repeated measurements in place at the parks. The change values to be detected are 2% for all indicators, except for non-indigenous plant propagation, which is 5%. These levels are commonly used in plant and animal monitoring studies [26],[27],[28]. The statistical power was calculated for these two change values to be detected for all study indicators. Concerning the degree of significance (α), 0.2 corresponds to the minimum acceptable in ecological monitoring [8], [25]. The calculation was also done with a degree of significance of 0.1 in order to evaluate the impact of this parameter on the statistical power. These two values were tested as they are commonly used in survey design optimization studies [26],[29],[30]. The average and standard deviation values for each sampling site were calculated using the field data collected since the implementation of PSIE, in 2004. The use of this type of data is still the best way to estimate these parameters [25],[31]. The values of the other input parameters come from the sampling protocols for each of the study indicators and the Monitor user guide.

## 3. Results

To reduce the length of the results section, only the detailed results for one park are presented for all three steps of the BIOPSIE approach (i.e. spatial characterization of ecosystems, ecological validation of indicators, statistical validation of indicators). Summary grids are presented for the other two parks.

### 3.1 Detailed results for the Frontenac National Park

#### 3.1.1 Spatial characterization of ecosystems

The park consists of three main ecosystems. The forest ecosystem dominates and covers 83.8% of the park’s area. The freshwater ecosystem covers 10.6% of the park’s area, and the wetland ecosystem covers 5.3% of the park. Even though it is not considered as an ecosystem in this project, the bare ground class was also mapped and represents 0.3% of the park. A detailed map of ecosystems is presented in S2 Fig.

The validation of the classification indicates an overall accuracy of 88% and a Kappa coefficient of 84%. These values, above the 80% threshold indicating an accurate classification [32], nevertheless have relatively substantial omission errors for the wetland (19.3%) and forest (16.5%) classes and commission errors for the bare ground (21.3%) and wetland (16.3%) classes (S2 Table). The wetland class was mainly confused with the forest class for the commission errors and with the three other classes for the omission errors. For its part, the forest class was confused with the wetland class for the omission errors. Finally, the commission errors for the bare ground class were linked with the freshwater and wetland classes.

#### 3.1.2 Ecological validation of indicators

**Fig 3:**
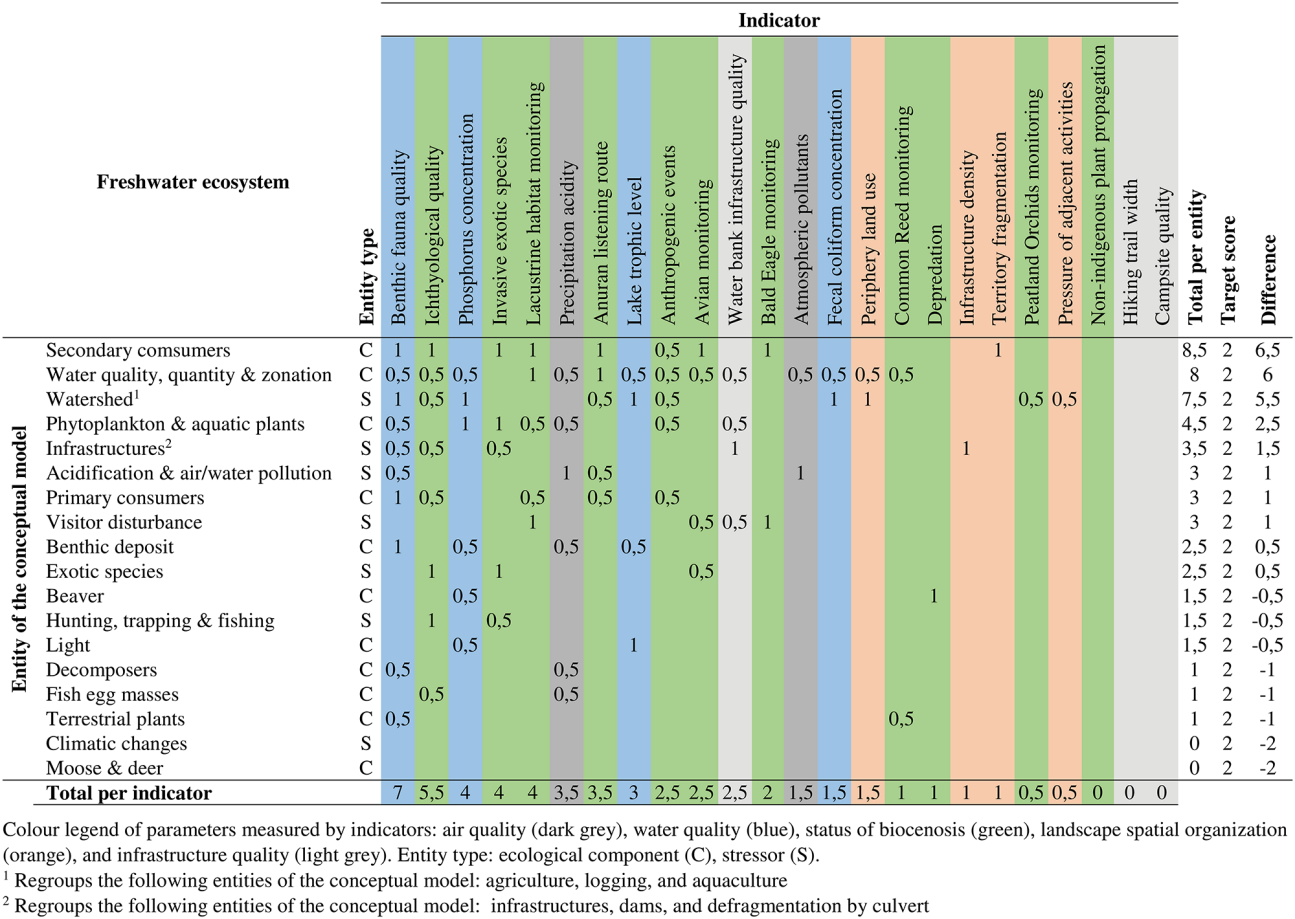
Detailed analysis grid of the ecological validation of indicators for the freshwater ecosystem of Frontenac National Park.

To illustrate the ecological validation of indicators, only the results related to the freshwater ecosystem are presented, in connection with the conceptual model presented in S1 Fig. The analysis grid for this ecosystem is presented in Fig 3.

Among the 18 entities linked to the freshwater ecosystem, 10 (5 ecological components and 5 stressors) are adequately covered by the indicators in place. Of these, the “Secondary consumers”, “Water quality, quantity & zonation” and “Watershed” entities are those with the highest scores with respective values of 6.5, 6 and 5.5. The “Secondary consumers” entity is directly measured by eight indicators, including those concerning the monitoring of selected fauna species or rare and endangered species. As for the “Water quality, quantity & zonation” entity, its score is explained by the fact that it is measured, often indirectly, by 14 of the park’s indicators. The other entities with a positive evaluation have values ranging from 0.5 to 2.5.

Among the eight entities that are not sufficiently measured, only two are stressors: “Climatic changes” with an evaluation of -2 and “Hunting trapping & fishing” with an evaluation of–0.5. Two other entities have an evaluation of -0.5: “Beaver” and “Light”. These entities are measured but have a score slightly less than the target score. The “Decomposers”, “Fish egg masses” and “Terrestrial plants” entities have an evaluation of -1. All three of them are indirectly measured by two indicators. Finally, the “Moose & Deer” entity has an evaluation of -2.

The indicators linked to the water quality parameter are those that provide the most information on the freshwater ecosystem. Of these, the fecal coliform concentration indicator has the lowest total. Furthermore, it measures entities (“Water quality, quantity & zonation” and “Watershed”) that are largely considered by other indicators. As for the indicators linked to the status of biocenosis, they have variable results depending on the monitored species (or group of species) and their relation with the freshwater ecosystem. For the two indicators linked to air quality, the precipitation acidity has the highest total. It allows an indirect monitoring of many entities that are sensitive to a variation in the environment’s acidity, including “Phytoplankton & aquatic plants” and “Decomposers”. The indicators linked to the human component have low total scores, except for the water bank infrastructure quality indicator.

This section primarily aimed to illustrate the explanatory potential of the detailed analysis grid. These are partial results since they represent only one of the three ecosystems in the park. A complete summary grid of all the park’s ecosystems is presented in section 4.1.4.

#### 3.1.3 Statistical validation of indicators

The 11 indicators in the study and their main sampling characteristics are presented in Table 1. The monitored permanent sampling sites were all chosen based on the judgment of park managers, except for campsite quality monitoring, for which the sites were chosen randomly.

Table 2 shows the results for the statistical power calculations for monitoring period values of 10 and 20 years for two detected change rates (2% and 5%) and two levels of significance (0.10 and 0.20). The distribution of indicators according to their statistical power and the various combinations of statistical parameters are summarized in Table 3. It is normal that the power is lower for a negative change; the more the population decreases, the more a change becomes difficult to detect [25]. The lowest power values were used to classify the indicator. For a period of 10 years, for an annual change of 2% (net negative change of 16.63% and positive change of 19.51%) and an α value of 0.10, the results show that the statistical power of the 11 indicators studied is low for 9 of them, medium for 1 and high for another (the lake trophic level indicator). In other words, it is the only indicator that will detect at least 80% of changes with a maximum of 10% error. Considering an α value of 0.20, the statistical power of campsite quality monitoring goes from low to medium. The changes are greater when varying the change parameter to be detected. Thus, for the same 10–year period (α = 0.10) and an annual change of 5% (net negative change of 36.98% and positive change of 55.13%), the statistical power is low for three indicators, medium for three others and high for the last five. For an α value of 0.20, two indicators (anuran listening routes and monitoring of a lacustrine habitat) go from medium to high power (Tables 2 and 3).

**Table 2:** Statistical power (%) for the Frontenac National Park indicators over two monitoring period (10 and 20 years), for two change rates to detect (2% and 5%) and two levels of significance (0,10 and 0,20).

	Power values (%) for monitoring periods of 10/20 years							
	α = 0,20				α = 0,10			
Indicator	-2%	2%	-5%	5%	-2%	2%	-5%	5%
Benthic fauna quality	18/42	20/70	44/91	73/100	8/25	10/53	25/79	52/100
Lake trophic level	98/100	100/100	100/100	100/100	96/100	100/100	100/100	100/100
Phosphorus concentration	21/24	22/33	24/45	38/100	10/14	11/21	14/32	25/100
Fecal coliform concentration	21/22	22/23	22/25	24/77	11/12	12/13	12/15	13/66
Non-indigenous plant propagation	78/100	90/100	100/100	100/100	67/100	83/100	100/100	100/100
Avian monitoring	48/93	59/100	97/100	99/100	34/88	45/100	95/100	100/100
Anuran listening route	30/82	37/100	80/100	100/100	18/71	23/99	70/100	99/100
Ichthyological quality	30/65	35/93	73/100	98/100	18/50	22/87	59/99	95/100
Lacustrine habitat monitoring	33/71	38/96	80/100	99/100	20/58	26/92	67/100	98/100
Hiking trail width	41/99	56/100	98/100	100/100	25/97	38/100	95/100	100/100
Campsite quality	51/100	65/100	100/100	100/100	36/100	52/100	99/100	100/100

**Table 3:** Distribution of indicators measured at the Frontenac National Park according to their statistical power calculated for different monitoring periods, change rates to detect, and levels of significance.

	Number of indicators		
	Low power [0–50%[	Medium power [50–80%[	High power [80–100%]
10 years, -2%, α = 0,20	8	2	1
10 years, -5%, α = 0,20	3	1	7
10 years, -2%, α = 0,10	9	1	1
10 years, -5%, α = 0,10	3	3	5
20 years, -2%, α = 0,20	3	2	6
20 years, -5%, α = 0,20	2	0	9
20 years, -2%, α = 0,10	3	3	5
20 years, -5%, α = 0,10	2	1	8

Considering a monitoring period of 20 years, instead of 10 years, results in a considerable increase in the statistical power of the indicators. For an annual change of 2% (net negative change of 31.88% and positive change of 45.68%) and an α value of 0.10, the power is low for three indicators, medium for three indicators and high for the five others. Considering instead an α value of 0.20, an indicator goes from a statistical power of medium to high. For an annual change of 5% (net negative change of 62.26% and positive change of 152.70%) and an α value of 0.10, two indicators have a low power, one has a medium power, and eight have a high power. Considering instead an α value of 0.20, the statistical power of the benthic fauna quality indicator goes from medium to high. Nevertheless, even considering permissive values of the significance level (α = 0.20), detected change (5%) and the monitoring period (20), phosphorus and fecal coliform concentration indicators remain problematic (Tables 2 and 3).

#### 3.1.4 Results summary of BIOPSIE approach

**Fig 4:**
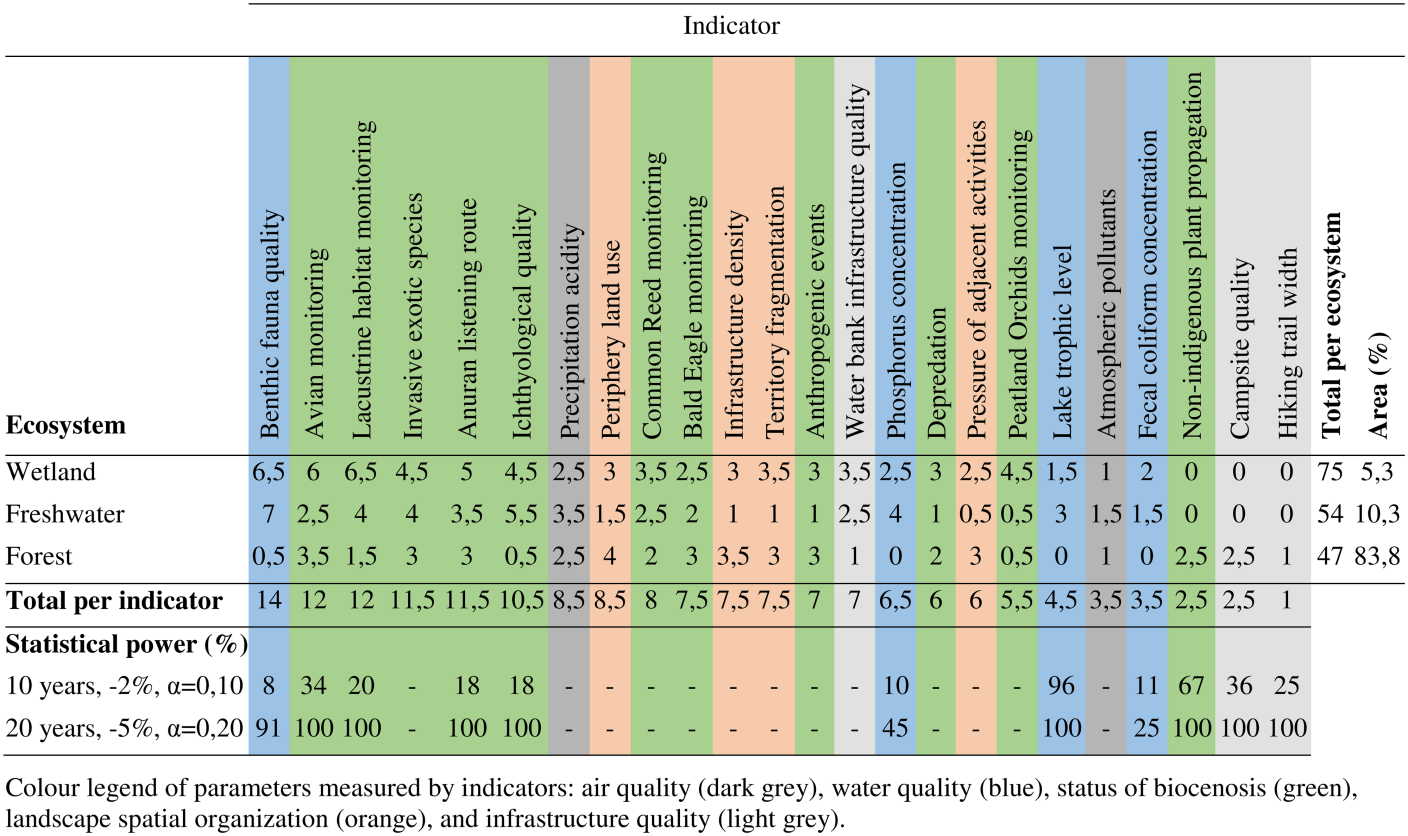
Summary analysis grid of the BIOPSIE for the Frontenac National Park.

The evaluation of indicators at the scale of the park involves analyzing information for all three ecosystems. Fig 4 summarizes the results for these three ecosystems. At the scale of the park, the benthic fauna quality indicator has the highest total (14), especially due to its high scores for freshwater and wetland ecosystems. Other indicators related to the water quality parameter have totals between 4 and 7 and are less suitable for all the park’s ecosystems. Five indicators have totals greater than or equal to 11: avian monitoring, lacustrine habitat monitoring, invasive exotic species, anuran listening route, and ichthyological quality index. They are all part of the status of biocenosis parameter and represent animal species covering the entities in the conceptual models of the park’s three ecosystems. The precipitation acidity indicator has a total of 9. This is mainly due to its ability to provide information on the freshwater ecosystem and terrestrial vegetation. The other indicator related to the air quality parameter, atmospheric pollutants, has a total of 4. It ranks among the worst indicators at the scale of the park. The indicator for pressures of adjacent activities has a total of 9. In this sense, it is one of the few to consider several stressors in the three conceptual models, for example hunting, trapping, and infrastructure. Other indicators related to the landscape spatial organization parameter rank in the mid range with totals between 6 and 8. The indicators related to the infrastructure quality parameter, except the water bank infrastructure quality indicator, are among those with the lowest totals.

The statistical power values calculated according to the most restrictive values (-2%, α = 0.10 and 10 years) and the least restrictive values are also shown (-5%, α = 0.20 and 20 years) in the summary grid in Fig 4. They help to appreciate the statistical significance of the indicators in addition to the ecological evaluation. For example, although the indicator for lacustrine habitat monitoring is ecologically very relevant for the park, the current sampling makes it difficult to derive scientifically sound information (power estimated at 34% for the most restrictive conditions). Improvements in the sampling protocol are required to use this indicator to its full potential.

The last two columns of Fig 4 show the relative importance of the park ecosystems relative to the total score of the indicators by ecosystem. For Frontenac Park, the wetland ecosystem is the one whose total is the highest (75). Yet it is the one with the smallest area (5.3%). Conversely, the forest ecosystem has a lower total (47) for a larger relative area (84% of the park).

### 3.2 Results summary for Jacques-Cartier national park

**Fig 5:**
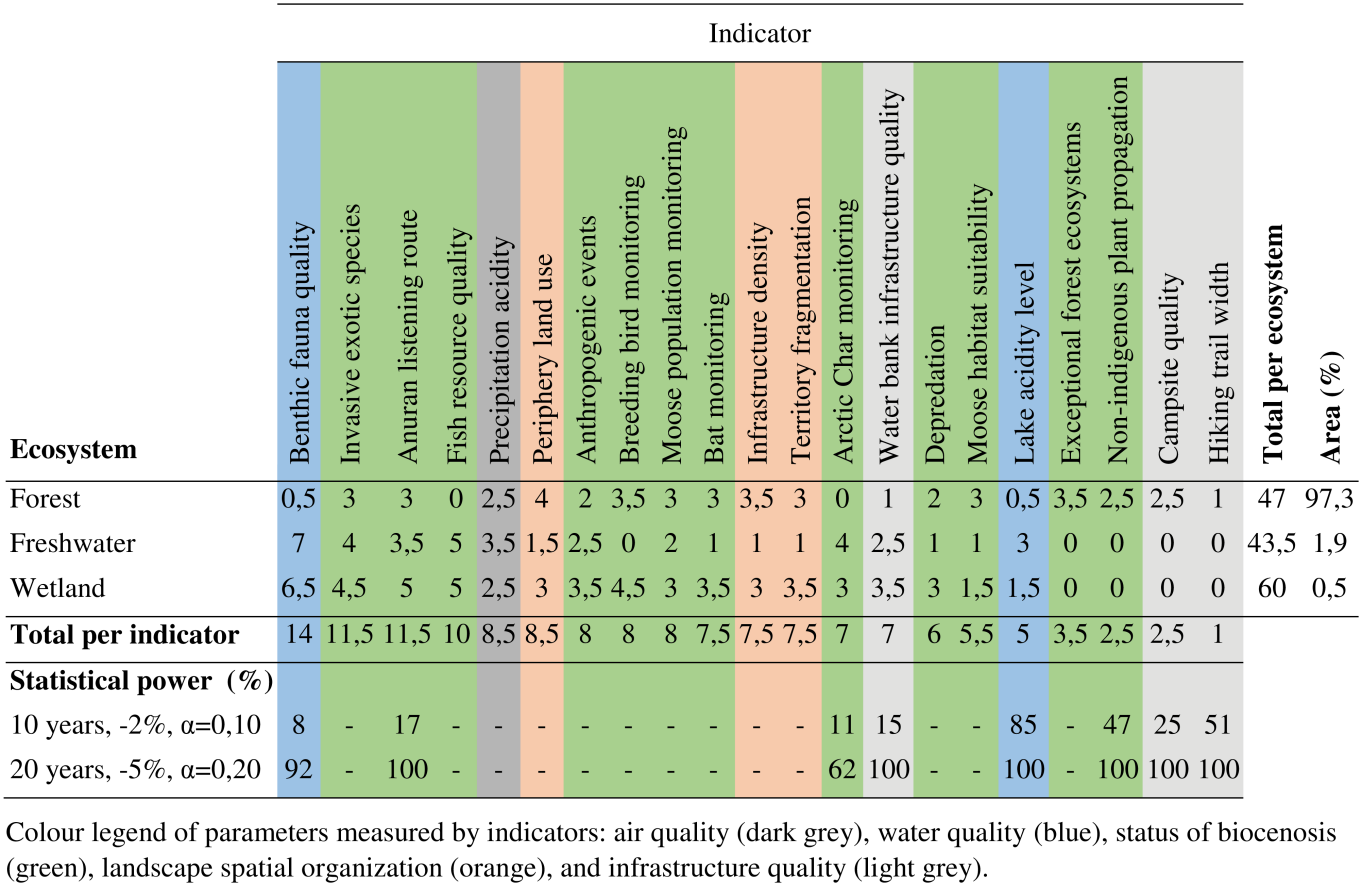
Summary analysis grid of the BIOPSIE for the Jacques-Cartier national park.

Fig 5 presents the results of the integration of the results for the three BIOPSIE methodological axes for Jacques-Cartier Park. At the scale of the park, the benthic fauna quality indicator, with a total of 14, has the highest score. This is mainly due to its contribution to freshwater and wetland ecosystems. The totals for other indicators range between 11.5 (exceptional forest ecosystems and anuran listening route) and 1 (hiking trail width). Indicators related to the status of biocenosis had varying scores depending on the monitored species. The indicators related to the landscape spatial organization had totals ranging between 7.5 and 8.5. As for Frontenac Park, indicators related to the infrastructure quality generally had low scores, except for the water bank infrastructure quality. Six indicators had a low or medium statistical power under restrictive conditions (-2%, α = 0.1 and 10 years) and high for permissive conditions (-5%, α = 0.2 and 20 years). For more permissive conditions the power for Arctic Char monitoring was medium (62%). Although the forest ecosystem covered more than 97% of the park’s area, it had a total score of 47 compared to 43.5 for the freshwater ecosystem and 60 for the wetland ecosystem.

### 3.3 Result summary for Bic National Park

**Fig 6:**
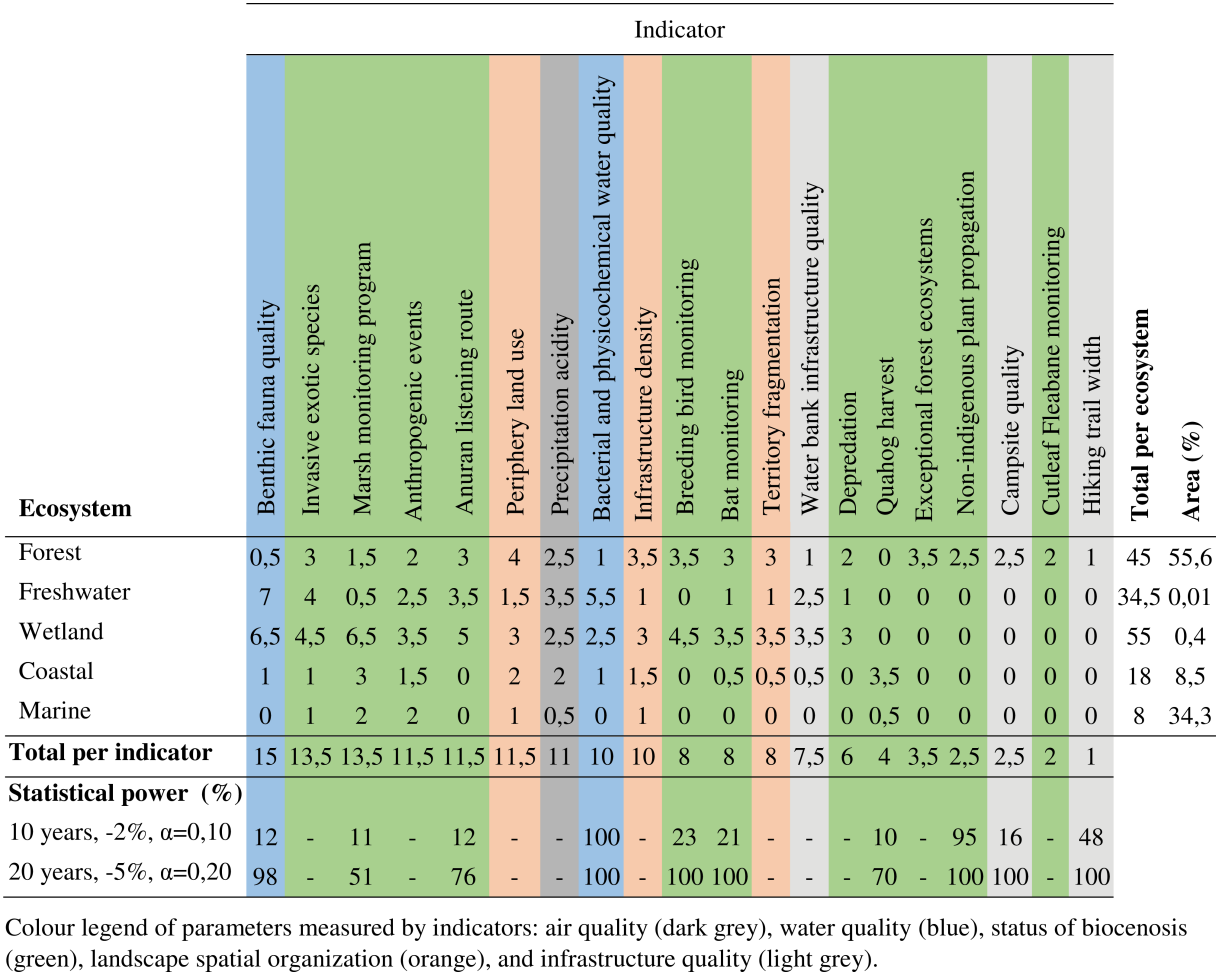
Summary analysis grid of the BIOPSIE for the Bic National Park.

Fig 6 presents the summary grid for Bic National Park, which is composed of five ecosystems. At the scale of the park, the benthic fauna quality indicator has the highest score (15). It measures the entities for both the park’s freshwater and wetland ecosystems. This result is similar to the two other parks in the study area. In total, the freshwater and wetland ecosystems, for which the indicator is especially efficient, cover less than 0.5% of the park’s area. The other indicators have total scores ranging between 13.5 and 1. Nine of the 20 indicators have a score higher or equal to 10. Among them, some measure entities in all of the park’s ecosystems. Anthropogenic events monitoring is an example. It has a score ranging between 1.5 and 3.5 for each of the five ecosystems. Its total of 11.5 is one of the highest for the indicator groups. The indicator for the anuran listening route is more specific. It also has a total score of 11.5, but it is divided among the freshwater, wetland, and forest ecosystems. Together, these ecosystems cover about 56% of the park’s area. Conversely, the quahog harvest indicator is specific to the coastal and marine ecosystems. Despite its relatively low total (4), it is the only one uniquely focused on these ecosystems.

Indicators of the landscape spatial organization had scores ranging between 8 and 11.5. They are efficient for the forest and wetland ecosystems. Indicators for the infrastructure quality, except the indicator of the water bank infrastructure quality (7.5), had low scores.

Considering the most restrictive sampling values, two indicators had a high statistical power (bacterial and physicochemical water quality index, and non-indigenous plant propagation). The power of the eight other indicators was low. Considering instead the least restrictive values, seven indicators had a high statistical power and three had a medium power.

The forest ecosystem is dominant (55.6% of the area). It had a total of 45 points, which is the second-highest score. Similar to the other two parks, the wetland ecosystem has the highest total. However, its area is limited (0.4%). The freshwater ecosystem also had a high score (34.5) despite an area less than 1%. Coastal (18) and marine ecosystems (8) had the lowest scores. Together, they cover almost 43% of the park’s area.

## 4. Discussion

### 4.1 Diagnosis and recommendations for the three studied parks

The BIOPSIE approach applied to the monitoring program for the three parks in the Quebec national parks network helped to develop a monitoring diagnostic and to identify specific improvements for monitoring. Due to space limitations, only one detailed analysis grid (Fig 3) for each ecosystem present in every park (11 detailed grids) is presented, as well as a single conceptual model (S1 Fig) of the ecosystems present in the three studied parks (5 conceptual models). However, in the optic of demonstrating the full potential of BIOPSIE, this section includes diagnostic elements and recommendations from the entire and detailed analysis of the three parks in the study (not included in the article).

First, the lack of coverage of soil related entities in the forest ecosystem is observed for the three tested parks. The entities concerned are “litter”, “humus & mineral soil”, and “decomposers”. Current scientific knowledge nevertheless indicates that soil is of great importance in the ecological integrity of terrestrial ecosystems [33]. Monitoring a soil-related indicator or an indicator species whose life cycle depends on it would improve the coverage of this ecosystem. For example, monitoring of soil acidity provides information on entities related to the ground, and those related to forest vegetation [34]. The total amount and concentration of carbon in the ground reflect most of the soil-related changes in forest ecosystems. Therefore, their monitoring provides a good understanding of the ecosystem while limiting the costs associated to a higher number of indicators [35]. An effort could thus be put into the creation of this type of indicators.

The results of the application of BIOPSIE also showed that the Climatic changes entity, present in all tested conceptual models, always gets a negative balance. In the better cases, it is indirectly measured by certain indicators, however, no indicator directly measures it. The implementation of a weather monitoring indicator is a possible solution, but it usually takes a minimum of 30 years of statistical data gathering on atmospheric parameters (rainfall, temperature, sunlight, humidity, etc.) before this information can be tied to a changing climate [36]. Regionalization of a climate model to the scale of a national park is also an important challenge. A possible solution is by modeling the apprehended evolution of monitored species’s ecological niche. These models link the observed distribution of a species with environmental variables such as climate, topography, and soil types [36].

The summary grids of the three parks also illustrate that infrastructure quality indicators usually get the lowest scores. According to the ecological monitoring method used by BIOPSIE, they therefore seem less relevant. A discussion on the bases of these indicators in the PSIE is to be expected. Their monitoring possibly provides more information on the visitor experience, who prefers trails and camping sites in good condition, than on the ecological integrity parks. Trail traffic might be a more appropriate measure to monitor the level of ecological integrity in parks. In this sense, several studies show a link between the presence of trails and visitors and that of certain animal species. This is the case for certain large mammals [37] and bird species [38]. Furthermore, the visitor disturbance entity is present in every conceptual model used in this project.

#### 4.1.1 Sampling site selection

The only randomly sampled indicators are “campsite quality” and “water bank infrastructure quality” (Jacques Cartier National Park only). In other words, the vast majority of monitored indicators are not subjected to probabilistic sampling. Therefore, the inference of the results to the entire park is not automatic as is the case for probability sampling [10]. The inherited data is too important to discard and it is hardly conceivable for park managers to change all the current sampling sites. For certain indicators, the same sites have been monitored for nearly 10 years. In this sense, [8] recognizes that this leads to the use of statistically inappropriate sites. However, sampling approaches such as Spatially Balanced Sampling (SBS) enables the integration of existing sampling sites. It allows stratifying the area to be sampled. This characteristic is particularly interesting in that it allows focusing the sampling efforts and thus decreases the costs, depending on the ecological and accessibility constraints, while also presenting a statistically defensible framework [39]. For example, freshwater ecosystem indicators could be classified using Strahler stream order in order to assign different weights on different categories of the monitored resource [40]. SBS sampling also allows integration of resource accessibility for sampling site selection. For example, travel-associated costs could be reduced by selecting sites based on their proximity to access roads. However, the choice of optimizing the sampling of an indicator, should be made carefully. This choice must be made in relation with all the existing indicators to take into account possible synergies (e.g. multiple indicators measured at a same site), or cost/benefit considerations (e.g. logistic impacts of increasing sampling sites over significance of improvement).

#### 4.1.2 Importance of change detection on statistical power

The statistical power analyses show that for nine of the evaluated indicators, a shift threshold of 5% instead of 2% leads to greater statistical power, even in demanding sampling situations. However, the Sépaq usually sets the threshold to 2% per year for most of the measured indicators, except for the non-native plants indicator which is of 5%. The literature review suggests that these values are too rigorous to be implemented. According to [25], a value of 2% is low and makes it difficult to achieve sufficient statistical power. In the context of ecological monitoring, a value of 5% is more realistic. A rise in threshold value might enable a majority of indicators to reach a high statistical power value. Discussion regarding the optimization of sampling protocols could then be directed towards the most problematic indicators.

#### 4.1.3 The monitoring period

The monitoring period, which is closely related to the importance of change detection, also has a major impact on statistical power evaluation. As part of this project, statistical power was calculated for fixed periods of 10 and 20 years. In some cases, results indicate that Sépaq managers may interpret the results over a period of 10 years. In most cases, however, results of the statistical analysis rather suggest a minimum follow-up period of 20 years. This longer period allows many indicators to obtain a high statistical power value. Conversely, it is also possible that the minimum follow-up period is less than 10 years, particularly in the case of annually sampled indicators. The reality is that the monitoring time depends on the measured indicator [8]. Natural abundance cycles of various animal species and steady growing plant species have different implications for the duration of monitoring. For example, [41] report a minimum of 10 years in the case of tracked fish to detect 80% of the changes in the population.

#### 4.1.4 Effect of variability on the statistical power

The variability values entered in Monitor for the statistical power calculations correspond to the standard deviation of the currently collected sample data. It corresponds to a total value of variation. However, it is possible to divide this variability into two types: process variation, which is linked to changes in the study population, and sampling variation, which results from the different possible measurement errors [25]. Considering a divided variance could allow a more accurate assessment of statistical power [25]. However, Sépaq seeks above all an overview of the indicator’s statistical power to guide the implementation of corrective actions where necessary. In this regard, BIOPSIE fulfills the mandate and allows the calculation of statistical power with sufficient precision for this management context.

When considering the most restrictive monitoring conditions, certain indicators still achieve a high statistical power. These indicators generally have low data variability (e.g. pH value). This single characteristic allows to reach high statistical power with a limited number of sites and low intra- and inter-annual revisit. Testing more restrictive thresholds of degrees of confidence on these indicators could lead to re-evaluate the possibility of reducing the sampling effort without affecting their statistical power. By contrast, the high variability of certain indicators prevents reaching sufficient statistical power (e.g. anuran listening route). In cases where the variability associated to monitoring cannot be sufficiently reduced, the relevance of this indicator could be doubted and that would eventually affect the interpretation of the results. In other cases, the expected variability should theoretically be low but is high in practice (e.g. campsite quality monitoring). The main consequence is to make it difficult to reach a high statistical power. This variability could be decreased by more precise instructions to the employees conducting these measurements. Ideally, a limited group of employees at a park should measure each of the indicators. This simple procedure would decrease the variability related to the observer.

### 4.2 Benefits and limitations of the BIOPSIE

Through spatial characterization of ecosystems, and ecological and statistical validation of indicators, the BIOPSIE has demonstrated its ability to diagnose the existing program and to identify potential optimization. These three steps each have their strengths and weaknesses.

#### 4.2.1 Ecosystem mapping

Although mapping does not allow direct assessment of an indicator’s relevance, it nevertheless helps to pursue their evaluation, according to ecological and statistical perspectives, through the information it provides on sampling location and ecosystem area. In this sense, it provides an avenue for sampling protocol improvement, even if territory accessibility limits may arise. It could be used as an input to a new sampling site selection. Mapping also provides managers with uniform landcover data for all the parks in the study area. In this sense, it is particularly important in the case of wetland class, whose cartographic data are often outdated or missing across Quebec. It also provides a standardized landcover layer that can be updated and integrated in temporal change analysis [42].

The resultant cartography data also puts the results of the environmental assessment into perspective. Combining the total score of ecosystem coverage to its relative area, allows to determine if the efforts dedicated to an ecosystem are sufficient. This approach is particularly revealing for Bic Park. It highlights the under-representation of coastal and marine ecosystem indicators, despite the relatively large size of these two ecosystems across the park.

Although ecosystem classification of the three parks has sufficient validation statistics, this one is subject to methodological limitations. Thus, the spatial resolution of SPOT 4 and 5 images (20 m) are not fine enough to allow mapping of medium and low importance rivers. This corresponds to the findings of [43], which highlight the fact that this type of entity is difficult to map for a sensor of this range of spatial resolution. In this case, the use of more precise auxiliary data is essential to a thorough knowledge of the mapped ecosystems.

#### 4.2.2 Simplified use of ecosystem conceptual models

The methodology developed fills a gap identified in the literature review. It allows to link the information contained in ecosystem conceptual models to a quantitative assessment of indicators. In this sense, it stands out from complex mathematical approaches suggested by [11] and [44] which are based on network analysis and graph theory. The quantitative evaluation of the BIOPSIE is innovative, as its quantitative aspect focuses on indicator contribution to measure conceptual model entities. It also highlights the fact that an indicator can measure more than one entity present in the model, a characteristic that is not featured in the literature review. The methodology does not allow fine use of the obtained quantitative values. However, the order of magnitude can be used by managers to support various improvement measures.

The ecological assessment method put forward with the BIOPSIE is also characterized by its flexibility. Possible changes in conceptual models (e.g. the addition of an entity) or the addition of an indicator, easily transposes into analysis grids. When the score of the new indicator or entity is calculated, it can be compared to the rest of the grid. This flexibility is desirable and consistent with the recommendation of [10] which states that the conceptual models should be evolving and reflect the latest ecosystem knowledge.

One of the criticisms often stated about conceptual models is that they are subjective and represent the views of their designer [35]. The use of models developed by Parks Canada specialists, within this project, aimed to reduce this subjectivity to a minimum. It still appears that the conceptual models provide only a simplified picture of interactions within an ecosystem. Some relationships may be absent or too complicated to be illustrated in a general model. For example, [20] mentions the effect of grazing on forest succession. It is then necessary to use a sub-conceptual model on a particular conservation issue of the park, to better understand the interactions.

#### 4.2.3 Analysis grids

Another issue related to the objectivity of this approach concerns the analysis grid content. The association of indicators to measured entities of the conceptual model is the basis for the indicator score and gains in rigor when several experts are involved. However, it remains difficult to confirm. When implanted in parks, evaluations will be carried out by several specialists who will use grids of well-defined criteria. This should decrease the subjectivity of the approach. The exercise performed is primarily intended to establish a relative assessment of the indicator’s ecological importance. The fine score value obtained by an indicator is not as important as their global positioning.

Some entities present in the analysis grids are general while others are very specific. The main consequence is reflected in the entities’ balance sheets. It is therefore easier for a general entity to obtain positive results.

For the three parks in the study, the wetland ecosystem is the one that gets the highest total score. The main reason is that the associated conceptual model contains entities present in both forest and freshwater ecosystems. This has the effect of increasing its own score. However, this does not necessarily mean that the ecosystem is well covered, and park managers should ensure that a minimum of PSIE indicators are specifically designed for this ecosystem.

The use of analysis grids allows the assessment of the contribution of individual indicators. It also quantifies the importance of the conceptual model’s entities and consequently directs the improvement of existing indicators and the choice of future indicators. However, its interpretation must be done wisely. Although certain indicators achieve a lower total score, the Orchids peatland in the Frontenac National Park for example, they remain very important to measure the changes in an ecosystem’s ecological integrity. This is usually the case of indicators that measure a very specific entity of the conceptual model. Indicator evaluation should be done by considering aspects other than the total score value obtained.

## 5. Conclusions

The BIOPSIE developed in this study provides a diagnostic tool to evaluate the ecological, spatial and statistical relevance of indicators from an existing ecological monitoring program. It provides a systematic, repeatable, and quantified method for protected area managers to diagnose the representativeness of ecological integrity indicators and to perform an iterative optimization process of their monitoring programs. It also provides a relatively simple and rigorous approach adapted to a wide variety of protected area networks, particularly to organizations with limited resources.

This tool has a multi-level application potential. Whether at the level of a protected area, an ecosystem (a park or network), a conceptual model entity (applied to a park or network) or an ecological integrity indicator (applied to a park or network), this tool can provide specific recommendations leading to optimized monitoring. It can also address the low representativity of certain indicators by increasing their statistical power (e.g. sampling optimization) or by identifying those needed to be replaced. At the protected areas network’s scale, this systematic approach also leads to the evaluation of an entire monitoring program and possibly to making adjustments on the entire network simultaneously.

The presented approach strengthens the capacity of protected area managers to implement a robust ecological integrity monitoring program. By entering into an optimization perspective, it allows to make corrections to existing programs without compromising the legacy of existing data.

## Supporting information

S1 Table Data source and characteristics. (DOCX)

S2 Table Error matrix describing classification results of ecosystems for the Frontenac National Park. (DOCX)

S1 Fig Conceptual model of freshwater ecosystem (adapted from Parks Canada, 2005). (DOCX)

S2 Fig Ecosystem mapping of the Frontenac National Park. (DOCX)
