# Supplementary material for: Optimization of an ecological integrity monitoring program for protected areas: Case study for a network of national parks
Source: PLoS One. 2018 Sep 19;13(9):e0202902. doi: 10.1371/journal.pone.0202902 (PMC6145595; doi:10.1371/journal.pone.0202902)
Supplement: S1 Table — (DOCX) [file pone.0202902.s001.docx]

**S1 Table. Data source and characteristics.**

| **Database** | **Format** | **Source** | **Scale/Resolution** | **Last update** |
| --- | --- | --- | --- | --- |
| Park limits and infrastructures | Vector | Sépaq | 1 : 20 000 | 2012 |
| Sampling sites | Vector & text | Sépaq | 1 : 20 000 | 2012 |
| BDTQ^1^ | Vector | Sépaq | 1 : 20 000 | 2011 |
| Aerial photographies | Raster | Sherbrooke University | 1 : 15 000 | 2007 - 2008 |
| Satellite imagery (SPOT 4 and 5) | Raster | GeoGratis^2^ | 10 & 20m | 2007 - 2008 |

^1^ Base de données topographiques du Québec (BDTQ) [computer file]. 1:20 000, Ministère des Ressources naturelles et de la Faune

^2^ GeoGratis [computer file]. Raster, Natural Resources Canada
