# Supplementary material for: Optimization of an ecological integrity monitoring program for protected areas: Case study for a network of national parks
Source: PLoS One. 2018 Sep 19;13(9):e0202902. doi: 10.1371/journal.pone.0202902 (PMC6145595; doi:10.1371/journal.pone.0202902)
Supplement: S2 Table — (DOCX) [file pone.0202902.s002.docx]

**S2 Table. Error matrix describing classification results of ecosystems for the Frontenac National Park.**

|  | Freshwater | Forest | Wetland | Bareground | Total |
| --- | --- | --- | --- | --- | --- |
| Freshwater | 76 | 0 | 4 | 0 | 80 |
| Forest | 0 | 76 | 5 | 0 | 81 |
| Wetland | 0 | 12 | 67 | 1 | 80 |
| Bare ground | 7 | 3 | 7 | 63 | 80 |
| Total | 83 | 91 | 83 | 64 | 321 |
| Omission error | 8,4 % | 16,5 % | 19,3 % | 1,6 % |  |
| Commission error | 5 % | 6,2 % | 16,3 % | 21,3 % |  |
| Overall acuracy | 88 % |  |  |  |  |
| Kappa coefficient | 84 % |  |  |  |  |
