# Supplementary material for: Optimization of an ecological integrity monitoring program for protected areas: Case study for a network of national parks
Source: PLoS One. 2018 Sep 19;13(9):e0202902. doi: 10.1371/journal.pone.0202902 (PMC6145595; doi:10.1371/journal.pone.0202902)

**S1 Fig. Conceptual model of freshwater ecosystem (adapted from Parks Canada, 2005). Stressors (red), natural disturbances (green), ecological components (grey), linking processes (blue) as well as solid lines (direct relationships between entities) and dotted lines (indirect relationships between entities) are represented.**


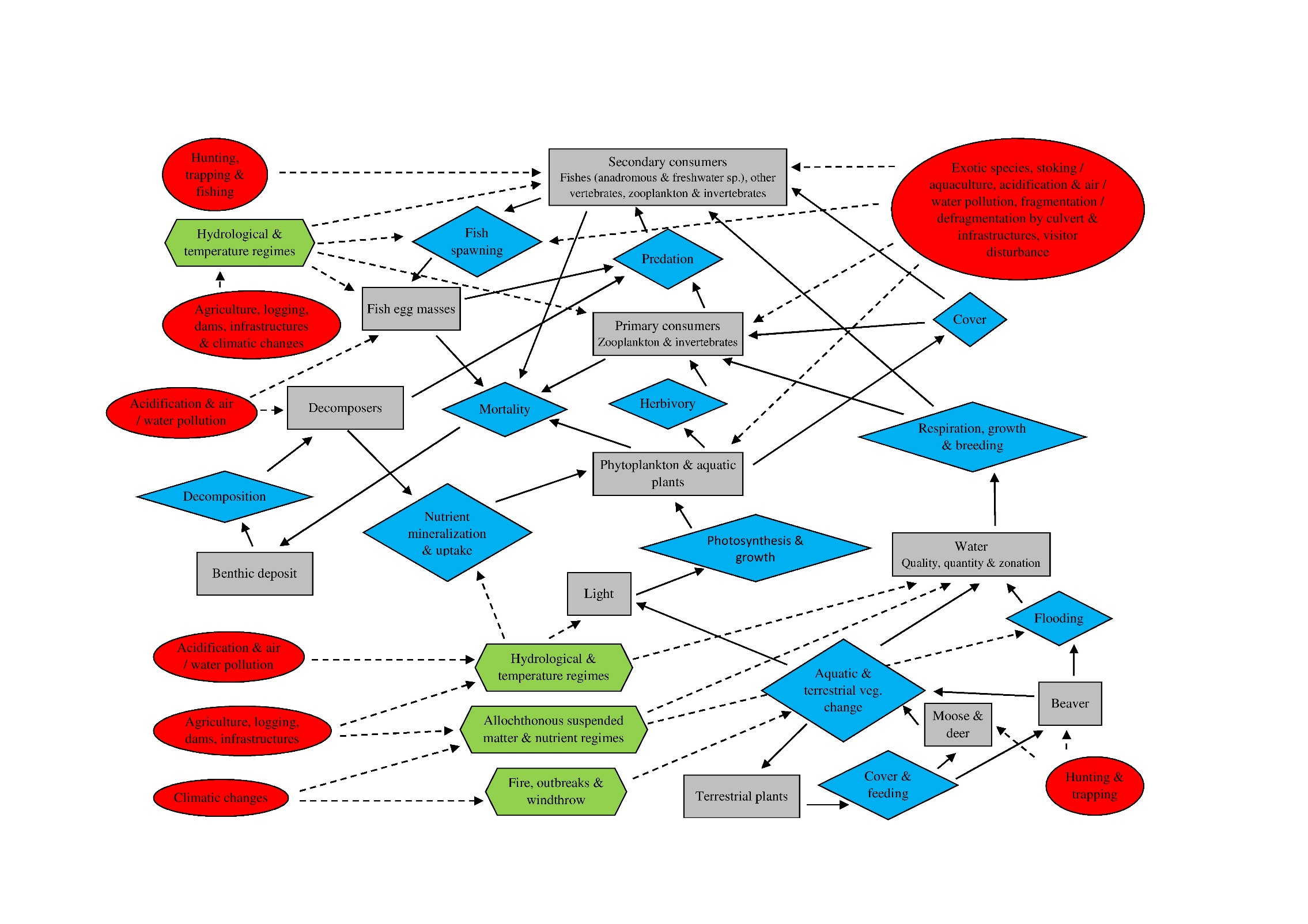

Supplement: S1 Fig — (DOCX) [file pone.0202902.s003.docx]
