# Supplementary figures and images for: Optimization of an ecological integrity monitoring program for protected areas: Case study for a network of national parks
Source: PLoS One. 2018 Sep 19;13(9):e0202902. doi: 10.1371/journal.pone.0202902 (PMC6145595; doi:10.1371/journal.pone.0202902)

**S2 Fig. Ecosystem mapping of the Frontenac National Park.**


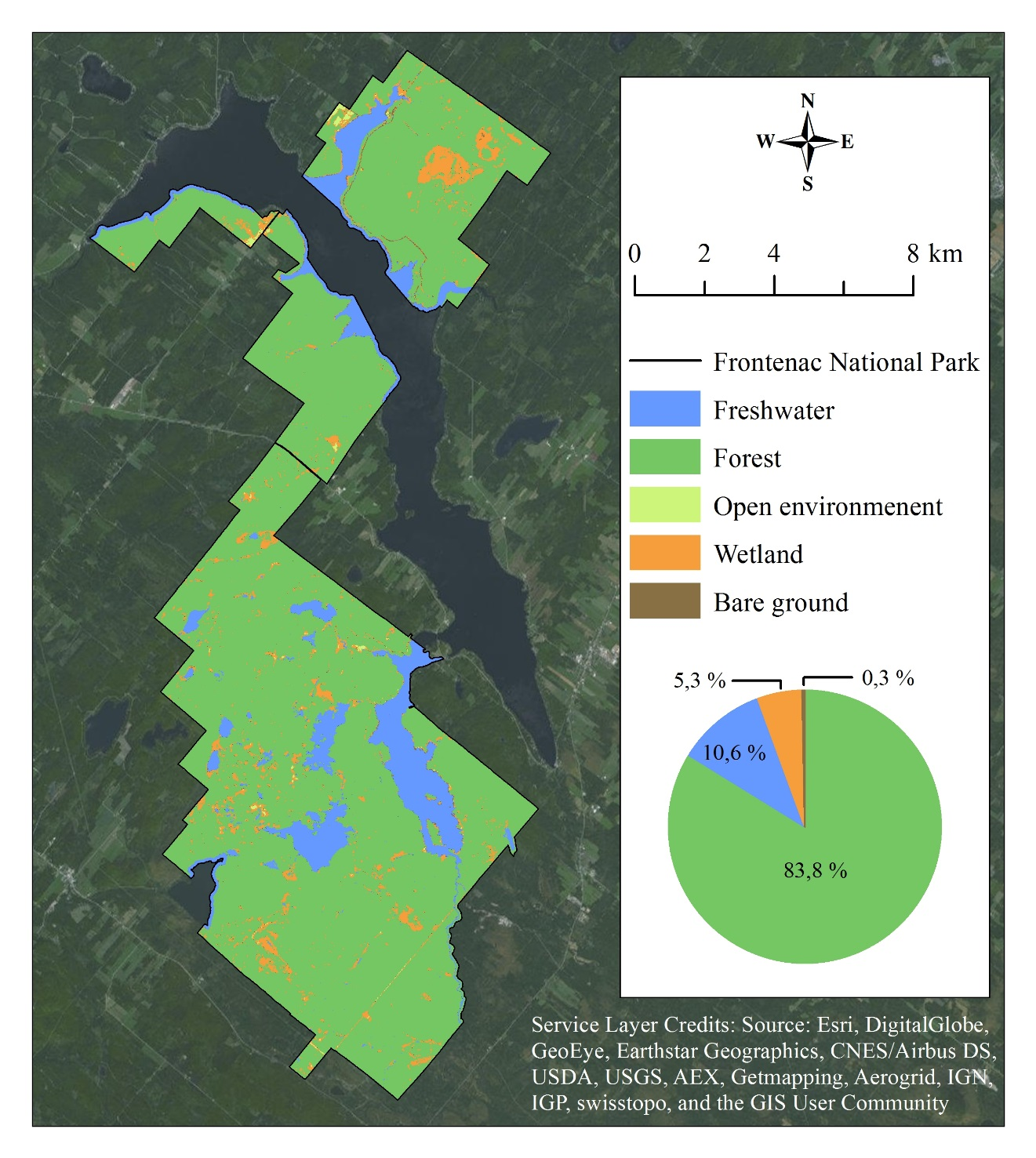

Supplement: S2 Fig — (DOCX) [file pone.0202902.s004.docx]
